# Lamotrigine-Associated Visual and Tactile Hallucinations in a Patient With Bipolar Disorder: A Case Report

**DOI:** 10.7759/cureus.116035

**Published:** 2026-09-10

**Authors:** Bárbara Santos, José Morais, Henrique Salgado

**Affiliations:** 1 Psychiatry, Hospital de São João, Porto, PRT

**Keywords:** adverse drug reaction, bipolar depression, bipolar disorder, lamotrigine, tactile hallucinations, visual hallucinations

## Abstract

Bipolar disorder is a chronic psychiatric illness characterized by recurrent episodes of mood disturbance and significant functional impairment. Bipolar depression is one of the most common and disabling manifestations of the disorder, contributing substantially to its overall disease burden. Lamotrigine is recommended as a first-line treatment for acute bipolar depression and for the prevention of depressive relapse. It is also recommended as an adjunctive strategy in combination with mood stabilizers for patients with persistent depressive symptoms. Despite its favorable tolerability profile, lamotrigine can be associated with adverse effects.

We describe a case of a patient with bipolar disorder who developed visual and tactile hallucinations after lamotrigine dose escalation, an uncommon but clinically relevant adverse drug reaction.

## Introduction

Bipolar disorder is a chronic psychiatric illness characterized by recurrent episodes of mania, hypomania, and depression, interspersed with periods of euthymia. It is associated with substantial morbidity, causing significant functional impairment and increased mortality, with suicide representing one of the leading causes of death in this population [1].

The management of bipolar disorder remains particularly challenging due to the recurrent nature of the illness and the marked heterogeneity of its clinical presentation. Although manic and hypomanic episodes are core diagnostic features, longitudinal studies have shown that the course of the disorder is frequently dominated by residual or recurrent depressive symptoms, which are associated with greater functional disability and poorer overall prognosis [2].

Over recent decades, the pharmacological management of bipolar disorder has progressively shifted from the sole prevention of manic episodes to treatment strategies targeting the full spectrum of the illness. In this context, major international guidelines recommend lamotrigine as a therapeutic option for bipolar disorder, because of its established role in the treatment of bipolar depression and, particularly, in the prevention of depressive recurrences throughout the course of the illness [1,3].

Lamotrigine is widely used in the management of bipolar disorder, particularly in the treatment of bipolar depression and prevention of depressive recurrence [1]. Evidence from randomized controlled trials indicates that lamotrigine provides a modest but consistent benefit in bipolar depression, particularly among patients with more severe depressive symptoms, while maintaining a favorable overall safety and tolerability profile [3]. Lamotrigine undergoes extensive hepatic metabolism, predominantly through glucuronidation mediated by UDP-glucuronosyltransferases [4]. Its pharmacokinetics are influenced by concomitant medications, with sodium valproate representing a clinically relevant inhibitor of lamotrigine metabolism that reduces its clearance and prolongs its elimination half-life, thereby increasing systemic exposure [5]. Risk factors for lamotrigine-associated psychotic symptoms remain poorly characterized. In a small case series of patients with epilepsy, psychiatric comorbidity and temporal lobe pathology were suggested as possible predisposing factors, although evidence remains limited [6]. Perceptual disturbances and psychotic symptoms have otherwise been reported only sporadically in the literature [6-10].

Uher and Jones reported the onset of visual hallucinations in a patient with bipolar disorder following an increase in the lamotrigine dose, with symptom improvement after dose reduction, suggesting a dose-dependent relationship [10]. Similarly, Hameed and Hamelijnck described a patient with bipolar disorder, without a history of epilepsy or psychosis, who developed visual and auditory hallucinations shortly after the initiation of lamotrigine treatment, with symptom exacerbation during dose titration and complete resolution following drug discontinuation [7]. More recently, Oh et al. reported a case of tactile hallucinations in a patient with bipolar II disorder, in whom symptoms resolved after lamotrigine discontinuation and recurred immediately following rechallenge, further supporting the plausibility of a causal association [8].

The available literature is limited to a small number of case reports. Consequently, the true frequency, predisposing factors, and underlying pathophysiological mechanisms of this adverse reaction remain poorly understood and are probably underrecognized [6-9].

In this case report, we describe the clinical features of a patient with bipolar disorder who developed visual and tactile hallucinations after an increase in the lamotrigine dose and discuss the clinical relevance of this potential adverse drug reaction in light of the currently available evidence. To our knowledge, only a very small number of cases describing concomitant visual and tactile hallucinations temporally associated with lamotrigine therapy have been reported, highlighting the importance of considering lamotrigine in the differential diagnosis of new-onset psychotic symptoms in patients with bipolar disorder.

## Case presentation

A 38-year-old Brazilian woman who had been living in Portugal for three months, with a several-year history of bipolar disorder, presented to the emergency department (ED) with a three-day history of acute visual and tactile hallucinations, described as “I see and feel worms everywhere,” accompanied by marked distress and behavioral changes. Her functional status was markedly impaired, with prominent psychomotor agitation, repetitive household cleaning and disinfection behaviors (including waking her children during the night to clean the house), markedly reduced oral intake, reduced fluid intake because of a “fear of infestation,” and associated insomnia.

The patient had been receiving remote psychiatric follow-up in Brazil and, at the time of presentation, was being treated with lamotrigine 100 mg/day, lithium 750 mg/day, and sodium valproate 1000 mg/day. Approximately three months before presentation, her treatment regimen had been progressively adjusted following a diagnosis of bipolar depression. Lithium had initially been gradually increased to 750 mg/day. Because of an inadequate clinical response, lamotrigine was introduced and gradually titrated in accordance with the recommended titration schedule for concomitant sodium valproate therapy. The dose was increased from 50 to 100 mg/day approximately two weeks before the onset of psychotic symptoms, while the sodium valproate dose was concurrently reduced from 1500 to 1000 mg/day.

She had no history of substance misuse and, when specifically questioned, denied recent use of recreational or synthetic psychoactive substances. Her medical history was also significant for attention-deficit/hyperactivity disorder (ADHD), for which she had been receiving lisdexamfetamine dimesylate 70 mg/day for the previous three years, with good tolerability and no previously reported adverse effects. The temporal relationship between pharmacological changes, symptom onset, and subsequent clinical evolution is summarized in Table 1.

**Table 1:** Timeline of pharmacological changes, symptom onset, and clinical course ED: emergency department

Time point	Pharmacological treatment and changes	Clinical course
~3 months prior to ED presentation	Treatment regimen progressively adjusted following a diagnosis of bipolar depression. Lithium gradually increased to 750 mg/day.	Inadequate clinical response.
Lamotrigine initiation and titration	Lamotrigine initiated and progressively titrated: 25 mg every other day (weeks 1-2), 25 mg/day (weeks 3-4), 50 mg/day (week 5), and 100 mg/day from week 6 onwards.	-
~2 weeks before symptom onset	Lamotrigine increased from 50 to 100 mg/day; sodium valproate concurrently reduced from 1500 to 1000 mg/day. Lisdexamfetamine remained unchanged at 70 mg/day (stable for approximately three years).	-
3 days before ED presentation	No further medication changes.	Acute onset of visual and tactile hallucinations (“worms everywhere”), marked distress, psychomotor agitation, repetitive cleaning and disinfection behaviors, reduced food and fluid intake, and insomnia.
ED presentation	At presentation to the ED, the patient was taking lamotrigine 100 mg/day, lithium 750 mg/day, sodium valproate 1000 mg/day, and lisdexamfetamine 70 mg/day. Olanzapine 5 mg/day was initiated during the ED visit.	Improvement in anxiety and reduction in hallucinations after olanzapine initiation.
At discharge	Lamotrigine reduced from 100 to 50 mg/day; lisdexamfetamine discontinued; lithium and sodium valproate continued; olanzapine 5 mg/day continued and subsequently increased to 10 mg/day.	Progressive clinical improvement.
~3 weeks after ED presentation	-	Complete resolution of visual and tactile hallucinations, euthymic mood, restored insight, normalization of sleep and appetite, and return to previous level of functioning.

On examination, the patient appeared well-groomed, alert, fully oriented, and cooperative. She appeared tense throughout the interview. Speech was spontaneous, fluent, and coherent, with no syntactic or semantic abnormalities, pressure of speech, or logorrhea. Her mood was anxious, with congruent affect. She reported visual and tactile hallucinations, with no other abnormalities in the form or content of thought. She denied suicidal ideation. She also reported poor oral intake and insomnia over the preceding days. Although she lacked insight into her perceptual disturbances, she agreed to treatment. Physical examination revealed a hemodynamically stable, afebrile patient with no respiratory distress and a peripheral oxygen saturation of 98% on room air. Cardiopulmonary auscultation was unremarkable. The abdomen was soft, non-distended, and non-tender on palpation. There was no peripheral edema or focal neurological deficit.

Given the acute psychotic presentation, a comprehensive diagnostic workup was performed. Complete blood count, renal function, electrolytes, liver function tests, folate, vitamin B12, total cholesterol, and blood glucose levels were all within normal limits. Serum valproate concentration was 103.1 mg/L (laboratory reference range: 50.0-100.0 mg/L; toxic range: >200.0 mg/L). Blood alcohol testing was negative, and urine toxicology screening was positive only for amphetamines, consistent with her prescribed lisdexamfetamine dimesylate treatment. Brain computed tomography (CT) revealed no acute abnormalities.

Olanzapine 5 mg/day was initiated in the ED, resulting in improvement of anxiety symptoms and a substantial reduction in hallucinations. The patient was discharged with instructions to reduce lamotrigine to 50 mg/day, discontinue lisdexamfetamine dimesylate, and continue olanzapine 5 mg/day, which was subsequently increased to 10 mg/day.

At outpatient follow-up approximately three weeks after the ED visit, she demonstrated complete resolution of visual and tactile hallucinations, with no residual psychotic symptoms. She had returned to a euthymic mood, denied suicidal ideation, had regained insight into the episode, and showed normalization of sleep and appetite, ultimately returning to her previous level of functioning.

## Discussion

This case report describes a patient with bipolar disorder who underwent multiple pharmacological adjustments following a depressive episode. Bipolar depression is among the most common and disabling manifestations of bipolar disorder and accounts for a substantial proportion of the overall disease burden [2]. Longitudinal studies have demonstrated that patients with bipolar disorder spend a considerable proportion of the illness course experiencing depressive symptoms, often exceeding the time spent with manic or hypomanic symptoms [2]. Beyond its temporal predominance, bipolar depression is associated with greater functional impairment, poorer quality of life, and an increased risk of suicidal behavior, making it one of the principal determinants of long-term prognosis [1,2].

Current recommendations acknowledge that many patients continue to experience residual depressive symptoms despite treatment with lithium or valproate [1]. In such cases, the addition of lamotrigine to an established mood stabilizer is an evidence-based therapeutic strategy recommended by major international guidelines [1,3].

Although evidence regarding the potential association between lamotrigine and visual or tactile hallucinations remains scarce, perceptual disturbances in our patient emerged after lamotrigine dose escalation and subsequently resolved following therapeutic intervention, which included lamotrigine dose reduction [6-10].

Initially, substance-induced and organic causes were considered. Visual and tactile hallucinations, particularly when occurring concurrently, warrant a thorough investigation for organic and substance-related etiologies. Formication, the sensation of insects crawling on or beneath the skin, is classically associated with stimulant intoxication, including cocaine and amphetamines [11]. Although standard urine toxicology screening does not exclude exposure to all synthetic psychoactive substances, the patient denied recent recreational or synthetic drug use when specifically questioned, and no clinical features suggestive of acute sympathomimetic intoxication were identified. Amphetamine-induced psychosis was also considered because the patient was receiving lisdexamfetamine 70 mg/day. However, the temporal pattern was less suggestive of a stimulant-induced reaction, as she had been receiving the same therapeutic dose for approximately three years without previous psychotic symptoms or recent dose adjustments. Nevertheless, given the recognized association between prescription amphetamines and psychotic symptoms, a contribution from lisdexamfetamine cannot be definitively excluded [12]. For this reason, lisdexamfetamine was discontinued as a precautionary measure during the acute psychotic episode. The positive urine toxicology result for amphetamines was consistent with her prescribed treatment and, in the absence of clinical features of acute sympathomimetic intoxication, did not provide evidence of stimulant intoxication. An organic etiology appeared less likely given the unremarkable laboratory investigations and normal cranial computed tomography findings.

Notably, similar clinical manifestations have previously been reported, although only rarely. Oh et al. described tactile hallucinations in the form of insects crawling on the skin, accompanied by repetitive checking behaviors, which resolved following lamotrigine discontinuation and recurred immediately after drug rechallenge [8]. Similarly, El Otmani et al. reported visual and tactile hallucinations, marked anxiety, and compulsive cleaning and washing behaviors after lamotrigine initiation, with symptom exacerbation after dose escalation and complete resolution after dose reduction and drug discontinuation [9].

The pathophysiological mechanism underlying lamotrigine-associated perceptual disturbances remains uncertain. At the cellular level, lamotrigine preferentially inhibits neuronal hyperexcitability through use- and voltage-dependent inhibition of neuronal voltage-gated sodium channels, thereby modulating synaptic transmission and reducing excessive neurotransmitter release [13]. However, a direct mechanistic link between these pharmacological effects and the development of hallucinations has not been established. Previous reports have proposed a possible intrinsic or toxic psychotogenic effect of lamotrigine, although the cellular and network mechanisms underlying this rare adverse reaction remain unknown [6].

An additional pharmacokinetic consideration is the concomitant use of sodium valproate, which inhibits lamotrigine glucuronidation, thereby reducing its clearance and increasing systemic exposure [5]. Although the valproate dose had been reduced from 1500 to 1000 mg/day during lamotrigine treatment, a clinically relevant pharmacokinetic interaction would still be expected [14]. The serum valproate concentration at presentation was 103.1 mg/L, marginally above the laboratory reference range but well below the toxic threshold. However, serum lamotrigine concentrations were not measured. Therefore, although concomitant valproate may have influenced lamotrigine pharmacokinetics, increased or supratherapeutic lamotrigine exposure could not be objectively demonstrated, and the contribution of this pharmacokinetic interaction to the development of the neuropsychiatric symptoms remains uncertain.

Application of the Naranjo Adverse Drug Reaction Probability Scale yielded a score of 5, indicating that the adverse reaction was classified as probable [15]. The individual items were assessed as follows: previous conclusive reports of similar reactions were identified (+1); the adverse event occurred after lamotrigine administration and dose escalation (+2); symptoms improved following lamotrigine dose reduction (+1); no rechallenge was performed (0); alternative causes could not be definitively excluded (-1); placebo was not administered (0); serum lamotrigine concentrations were not measured (0); the temporal relationship between dose escalation and symptom onset, followed by improvement after dose reduction, supported a dose-response relationship (+1); there was no known history of a similar reaction to lamotrigine or related drugs (0); and the adverse event was clinically documented during psychiatric assessment in the emergency department (+1). The resulting score of 5 supports a probable, rather than definite, association between lamotrigine and the reported perceptual disturbances.

An affective episode with psychotic features also remains a potential alternative explanation for the perceptual disturbances. However, the abrupt onset of symptoms, their close temporal relationship with lamotrigine dose escalation, and the absence of typical manic symptoms make this interpretation less likely.

A definitive causal interpretation is limited by the concurrent therapeutic interventions. Lamotrigine dose reduction occurred alongside discontinuation of lisdexamfetamine and initiation of olanzapine, both of which may have contributed to symptom resolution; notably, hallucinations had already substantially improved shortly after olanzapine initiation. The absence of serum lamotrigine concentrations and a rechallenge further limits causal inference. Nevertheless, the close temporal relationship between lamotrigine dose escalation and symptom onset, the probable association according to the Naranjo Scale, and the similarity to previously reported cases support an association between lamotrigine and the observed perceptual disturbances, without establishing a definitive causal relationship. This case therefore adds to the limited evidence that perceptual disturbances may emerge during lamotrigine treatment and highlights the importance of considering medication-related effects when new-onset psychotic symptoms occur after treatment initiation or dose escalation.

## Conclusions

Visual and tactile hallucinations are uncommon and poorly characterized adverse events reported in association with lamotrigine treatment. This case highlights a possible association between lamotrigine and the onset of hallucinations. Although a causal relationship cannot be established due to potential confounding factors and the clinical complexity of the case, reporting such observations may contribute to greater awareness of this potential adverse effect. Clinicians should be aware of this potential adverse reaction and consider lamotrigine, as well as clinically relevant pharmacokinetic interactions, when evaluating new-onset perceptual disturbances occurring during treatment, particularly following dose escalation. Further reports are needed to better characterize the frequency, underlying mechanisms, and potential predisposing factors associated with these uncommon neuropsychiatric manifestations.
